# Risk Factors for Human Brucellosis in Northern Tanzania

**DOI:** 10.4269/ajtmh.17-0125

**Published:** 2017-12-11

**Authors:** Shama Cash-Goldwasser, Michael J. Maze, Matthew P. Rubach, Holly M. Biggs, Robyn A. Stoddard, Katrina J. Sharples, Jo E. B. Halliday, Sarah Cleaveland, Michael C. Shand, Blandina T. Mmbaga, Charles Muiruri, Wilbrod Saganda, Bingileki F. Lwezaula, Rudovick R. Kazwala, Venance P. Maro, John A. Crump

**Affiliations:** 1 Duke Global Health Institute, Duke University, Durham, North Carolina;; 2 Kilimanjaro Christian Medical Centre, Moshi, Tanzania;; 3 Centre for International Health, University of Otago, Dunedin, New Zealand;; 4 Division of Infectious Diseases, Duke University Medical Center, Durham, North Carolina;; 5 Centers for Disease Control and Prevention, Bacterial Special Pathogens Branch, Atlanta, Georgia;; 6 Department of Mathematics and Statistics, University of Otago, Dunedin, New Zealand;; 7 Department of Medicine, University of Otago, Dunedin, New Zealand;; 8 Boyd Orr Centre for Population and Ecosystem Health, Institute of Biodiversity, Animal Health and Comparative Medicine, University of Glasgow, Glasgow, United Kingdom;; 9 Kilimanjaro Christian Medical University College, Moshi, Tanzania;; 10 Mawenzi Regional Referral Hospital, Moshi,Tanzania;; 11 Department of Veterinary Medicine and Public Health, Sokoine University of Agriculture, Morogoro, Tanzania

## Abstract

Little is known about the epidemiology of human brucellosis in sub-Saharan Africa. This hampers prevention and control efforts at the individual and population levels. To evaluate risk factors for brucellosis in northern Tanzania, we conducted a study of patients presenting with fever to two hospitals in Moshi, Tanzania. Serum taken at enrollment and at 4–6 week follow-up was tested by *Brucella* microagglutination test. Among participants with a clinically compatible illness, confirmed brucellosis cases were defined as having a ≥ 4-fold rise in agglutination titer between paired sera or a blood culture positive for *Brucella* spp., and probable brucellosis cases were defined as having a single reciprocal titer ≥ 160. Controls had reciprocal titers < 20 in paired sera. We collected demographic and clinical information and administered a risk factor questionnaire. Of 562 participants in the analysis, 50 (8.9%) had confirmed or probable brucellosis. Multivariable analysis showed that risk factors for brucellosis included assisting goat or sheep births (Odds ratio [OR] 5.9, 95% confidence interval [CI] 1.4, 24.6) and having contact with cattle (OR 1.2, 95% CI 1.0, 1.4). Consuming boiled or pasteurized dairy products was protective against brucellosis (OR 0.12, 95% CI 0.02, 0.93). No participants received a clinical diagnosis of brucellosis from their healthcare providers. The under-recognition of brucellosis by healthcare workers could be addressed with clinician education and better access to brucellosis diagnostic tests. Interventions focused on protecting livestock keepers, especially those who assist goat or sheep births, are needed.

## INTRODUCTION

Human brucellosis is a major zoonosis worldwide.^1,2^ It presents as an acute febrile illness^3,4^ and sometimes progresses to chronic debilitating disease.^5^ In addition to direct impacts on human health, brucellosis is associated with reproductive failure in domestic animals, resulting in economic losses for communities that rely on livestock for their livelihoods.^6,7^

Prevention of human brucellosis hinges on disease control in the livestock reservoir.^1^ Livestock vaccination and test and slaughter programs have been used in some countries to achieve elimination of both livestock and human brucellosis.^1,8,9^ However, such approaches have rarely been used in sub-Saharan Africa because implementation resources are scarce and the case for investment has not been made.^2,7^ In addition, the *Brucella* and livestock species that drive brucellosis epidemiology in sub-Saharan Africa remain unknown.^9^ While *Brucella melitensis* typically infects goats and sheep, and *Brucella abortus* typically infects cattle, cross-species infections have complicated control efforts in some places.^6^

*Brucella* localizes to the reproductive tract and mammary glands of livestock and may be present in the blood, reproductive tract secretions, and milk.^10^ Humans may acquire the infection through direct contact, foodborne transmission, or airborne transmission if an infectious source is aerosolized.^11^ Risk factors for human brucellosis vary by context because of different animal reservoirs and behavioral practices. For example, brucellosis has been associated with consumption of camel milk in Israel,^12^ with slaughtering pigs in the United States,^13^ and with exposure to livestock placentas in Chad.^14^

Brucellosis transmission to humans can be interrupted through behavior change, provision of personal protective equipment that limits human exposure to infectious sources, and food safety interventions that target meat or milk production.^1^ Such interventions rely on knowledge of the burden of brucellosis and specific local risk factors. A study on the etiology of febrile illness among hospitalized patients in northern Tanzania showed that 16 (3.5%) of 453 participants had confirmed acute brucellosis.^15^ The prevalence of antibodies against *Brucella* species among abattoir workers in northern Tanzania was 8%.^16^ Human brucellosis in Tanzania has been associated with assisting livestock births,^17^ and *Brucella* antibody seropositivity has been associated with slaughtering livestock,^16^ and with milking, herding, or assisting cattle births.^18^ These data imply that brucellosis is endemic in northern Tanzania and that exposure may occur through a range of livestock-oriented activities.

To identify locally appropriate prevention measures, more data on risk factors for active human disease, rather than for *Brucella* antibody seropositivity, are needed. In addition, it is necessary to determine which specific activities involving which livestock species are associated with greatest risk. To investigate risk factors for human brucellosis in northern Tanzania, we conducted a prospective cohort study of febrile patients using a rigorous brucellosis case definition and a detailed risk factor questionnaire.

## MATERIALS AND METHODS

### Study setting.

We conducted our study at two hospitals in Moshi, Tanzania. Mawenzi Regional Referral Hospital (MRRH) is a 210-bed regional hospital serving the Kilimanjaro Region. Kilimanjaro Christian Medical Center (KCMC) is a 450-bed consultant referral hospital that serves a large catchment area, including the Kilimanjaro Region and other regions, in northern Tanzania. Moshi (population > 180,000) is the administrative center of Kilimanjaro Region (population > 1.6 million)^19^ and is situated at an elevation of approximately 890 meters above sea level. The climate is tropical, with rainy seasons from October through December and from March through May. The Kilimanjaro Region is predominately rural.^19^ Agriculture in northern Tanzania is characterized by pastoralism and by a mix of smallholder systems involving mixed crop and livestock farming.^20^

### Study procedures and participants.

We enrolled pediatric and adult patients presenting to MRRH and KCMC from February 2012 through May 2014. On weekdays, we screened all patients admitted within the past 24 hours to the adult and pediatric medical wards at MRRH and to the adult medical ward at KCMC, as well as patients presenting to the outpatient department at MRRH. We enrolled consecutive eligible inpatients and every second eligible outpatient. Inpatients and outpatients were eligible to participate if they had an axillary temperature of > 37.5°C, or a tympanic, oral, or rectal temperature of ≥ 38.0°C at presentation. Inpatients were also eligible if they reported a history of fever within the past 72 hours. After obtaining informed consent, a trained study team member collected demographic and clinical information and administered a standardized risk factor questionnaire. The risk factor questionnaire included questions on dietary practices and daily activities performed within the past month, with a focus on agricultural and animal-related activities. Blood was drawn for aerobic blood culture, examination for blood parasites, and acute serum archiving. Healthcare workers who were not part of the study team delivered outpatient and inpatient care according to local hospital standards. Clinical diagnoses at patient discharge were recorded. We immediately communicated the results of critical laboratory tests, including blood parasite smears and blood cultures to responsible medical personnel. The *Brucella* serology results were provided once available. Participants were asked to return 4–6 weeks after enrollment for collection of a convalescent serum sample. Study personnel visited participant households to collect Global Positioning System (GPS) coordinates of the households.

### Laboratory methods and case definitions.

Blood cultures were performed using BacT/ALERT pediatric fastidious bottles for children or standard aerobic bottles for adults, which were loaded into the BacT/ALERT 3D Microbial Detection system (BioMerieux Inc., Durham, NC) and incubated for 5 days. Standard methods were used for identifying bloodstream isolates.^3,21^ Thick and thin blood smears were examined for blood parasites by certified laboratory technologists. We sent acute and convalescent serum samples to the US Centers for Disease Control and Prevention (CDC) for analysis using the *Brucella* microagglutination test (BMAT). Standardized *B. abortus* strain 1119-3 killed antigen (National Veterinary Services Laboratory, Ames, IA) was used for BMAT at a 1:25 working dilution. Results were read on a Scienceware Plate Reader (Bel-Art Products, Wayne, NJ). Minor modifications were made to the CDC’s standard BMAT, including the use of U-bottom plates, incubation at 26°C, and discontinued use of safranin.^22^

We defined brucellosis as a clinically compatible illness plus laboratory evidence of infection. For confirmed cases, laboratory evidence was either a ≥ 4-fold rise in *Brucella* antibody titer between acute and convalescent serum samples or a blood culture positive for *Brucella* spp. For probable cases, laboratory evidence was a single reciprocal titer ≥ 160.^23^ In the analysis, we pooled participants who met criteria for confirmed or probable brucellosis and classified them as brucellosis cases. We classified participants with reciprocal titers < 20 in both acute and convalescent serum samples as controls. Once BMAT results were available, the study team attempted to trace participants who met our brucellosis case definition so that untreated brucellosis could be managed.

### Geospatial data and definitions.

Population data were obtained from the National Bureau of Statistics, Tanzania. We grouped participants with household GPS data according to population density: urban zones had a population density of ≥ 1,000 inhabitants/km^2^, peri-urban zones had a population density of ≥ 300 inhabitants/km^2^ and were ≤ 15 km distance from urban zones, and rural zones had a population density of < 300 inhabitants/km^2^ or were > 15 km distance from urban zones. Population density was calculated from the 2012 Tanzania Population and Housing Census.^19^

### Statistical analysis.

Data were entered using the Cardiff Teleform system (Cardiff Inc., Vista, CA) into an Access database (Microsoft Corporation, Redmond, WA). Geospatial data were managed using QGIS, version 2.12.0 (Free Software Foundation, Boston, MA). Analyses were performed using STATA, version 13.1 (STATA-Corp, College Station, TX). The spatial scan statistic was performed using a Bernoulli model to assess for evidence of spatial clustering among cases using SatScan version 9.0 (www.satscan.org). We derived a socioeconomic status scale using principal components analysis.^24^

Because of the high ratio of independent variables to cases and resultant instability of our multivariable models, we combined independent variables to reduce the number in the analysis. We used a method of variable aggregation that also allowed us to quantitatively measure participant exposure to potential risk factors for brucellosis. We already had single variables that represented participant dairy exposure and participant livestock birthing exposure, so we created aggregated variables or exposure scales, to measure participant livestock blood exposure and participant livestock contact. We used an analytic hierarchy process to develop these exposure scales.^25^ First, we identified relevant behaviors and living conditions from the risk factor questionnaire to be included in each scale. We then identified locally experienced subject matter experts, including epidemiologists, livestock workers, physicians, and veterinarians. We asked experts to rank every behavior in a particular scale against all other behaviors in that scale, in terms of the likelihood of livestock blood exposure or the likelihood of livestock contact, using a nine-point bidirectional scale. We then combined the rankings to obtain each variable’s weight.^26^ To derive the consensus weight of each behavior, we calculated the geometric mean of the experts’ weights. We included only the weights assigned by experts who provided internally consistent answers, defined as achieving a consistency ratio < 0.2.^26^ To aid interpretation of exposure scores, we scaled the weights so that the minimum possible score on each scale was 0 and the maximum possible score was 20. For both the livestock blood exposure scale and the livestock contact scale, we produced versions for cattle, goats, pigs, and sheep in aggregate, for cattle alone, for pigs alone, and for goats and sheep together. Finally, we derived a score for each participant on each exposure scale depending on the reported frequency of relevant behaviors in the questionnaire. For example, if someone performed none of the activities in the cattle blood exposure scale, they would score “0” on that scale, and if they performed every activity in the sheep blood exposure scale, they would score “20” on that scale.

Participants who did not meet the definition of either a brucellosis case or a control were excluded from the analyses. Univariable logistic regression was performed to explore associations between potential risk factors and risk of brucellosis. The models grouped goats and sheep together. Odds ratios (ORs) and 95% confidence intervals (CIs) were reported when appropriate. We built a multivariable model to examine associations between multiple risk factors and odds of brucellosis. Decisions to include variables in the model were based on known or suspected associations with brucellosis, and both individual behaviors and exposure scales were included. The forms of the relationships between the exposure scales and brucellosis risk were determined using fractional polynomial models. Backward selection guided by the Akaike information criterion was used to arrive at a final model. All *P* values were two sided, and *P* < 0.05 was considered statistically significant.

### Research ethics.

Written informed consent was obtained from all adult participants and from the parents or legal guardians of minors. This study was approved by the KCMC Research Ethics Committee, the Tanzania National Institute for Medical Research National Research Ethics Coordinating Committee, an Institutional Review Board of Duke University Health System, the University of Otago Human Ethics Committee (Health), and the US CDC.

## RESULTS

### Participant enrollment and characteristics.

**Figure 1.**
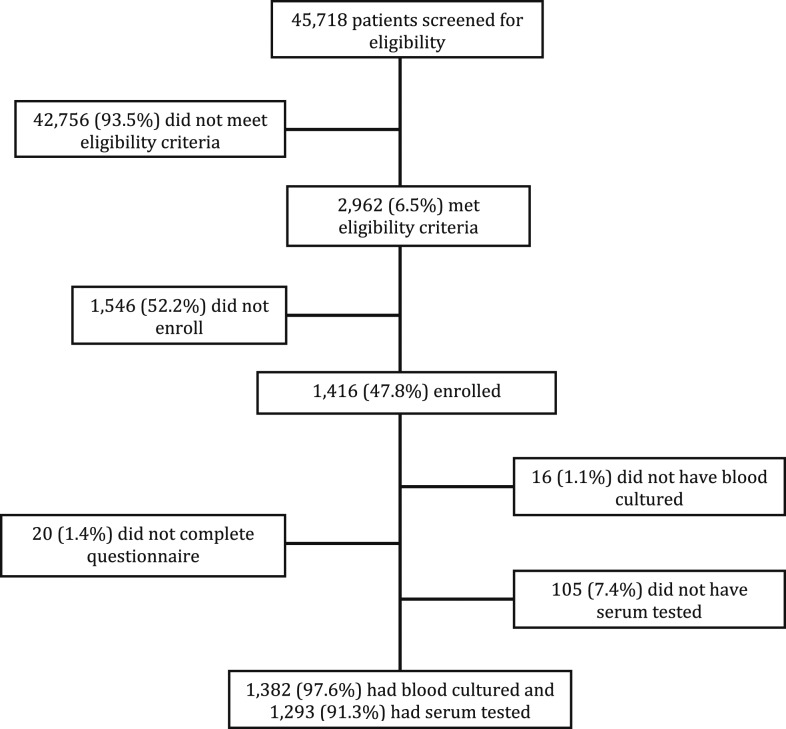
Study flow diagram for patients seeking care at Kilimanjaro Christian Medical Center and Mawenzi Regional Hospital in Moshi, Tanzania, 2012–2014.

Participant enrollment is summarized in Figure 1. Of 1,382 participants who had blood cultured, 63 (4.6%) grew pathogenic species but none grew *Brucella* spp. Of 1,293 participants who had serum tested for *Brucella* antibodies, 731 (56.5%) were excluded from the analysis because they did not meet brucellosis case or control definitions, 292 (22.3%) had an antibody titer ≥ 20 and < 160 in at least one serum sample, 439 (33.5%) had a titer < 20 in one serum sample but were missing a second sample, and 562 (43.5%) were included in the analysis. Of the 562 included in the analysis, 50 (8.9%) had brucellosis; 39 (6.9%) were confirmed cases and 11 (2.0%) were probable cases.

Participant demographic and clinical characteristics, and associated univariable regression results, are presented in Table 1. No participant received a clinical diagnosis of brucellosis. One hundred and twenty-nine (23.0%) of 562 participants received a clinical diagnosis of malaria, whereas 13 (2.3%) of 556 participants with available blood microscopy results had laboratory-confirmed malaria. Most participants had multiple presenting complaints in addition to fever.

**Table 1:** Demographic and clinical characteristics of participants with and without brucellosis, northern Tanzania, 2012–2014

	With brucellosis (*N* = 50)*		Without brucellosis (*N* = 512)*	
	*n*	(%)	*n*	(%)	OR (95% CI)	*P* value
Demographics						
Age, median (range) years	30.57 (0.57, 77.18)	n/a	20.55 (0.22, 93.5)	n/a	1.6 (1.2, 2.1)	0.001
Female sex	33	(66.0)	273	(53.3)	1.7 (0.89, 3.3)	0.115
Occupation†						
Butcher	0	(0.0)	3	(0.6)	–	–
Farmer	15	(30.0)	90	(17.6)	2.0 (1.0, 4.0)	0.060
Livestock attendant	1	(2.0)	7	(1.4)	1.8 (0.53, 5.2)	0.352
Vet	0	(0.0)	1	(0.2)	–	–
Other‡	34	(68.0)	410	(80.1)	0.53 (0.27, 1.1)	0.077
Pastoralist tribe§	0	(0.0)	8	(3.9)	–	–
Population density category						
Rural	11	(23.9)	109	(25.5)	n/a	n/a
Peri-urban	10	(21.7)	87	(20.3)	1.1 (0.46, 2.8)	0.777
Urban	25	(54.3)	232	(54.2)	1.0 (0.51, 2.2)	0.863
Residence in Moshi Urban District	28	(56.0)	245	(47.9)	0.75 (0.40, 1.4)	0.419
Socioeconomic status						
Lowest 25th percentile¶	18	(36.0)	119	(23.2)	n/a	n/a
Middle 50th percentile	19	(38.0)	264	(51.6)	0.48 (0.24, 0.94)	0.032
Highest 75th percentile	13	(26.0)	129	(25.2)	0.67 (0.31, 1.4)	0.292
Clinical history†						
Gastrointestinal symptoms	47	(94.0)	459	(89.6)	1.8 (0.55, 9.4)	0.480
Musculoskeletal symptoms	39	(78.0)	297	(58.0)	2.6 (1.3, 5.7)	0.007
Neurologic symptoms	44	(88.0)	330	(64.5)	4.03 (1.7, 11.8)	0.001
Respiratory symptoms	26	(52.0)	338	(66.0)	0.56 (0.30, 1.0)	0.071
Diagnosis†						
Clinical diagnosis brucellosis	0	(0.0)	0	(0.0)	–	–
Clinical diagnosis malaria	13	(26.0)	116	(22.7)	1.2 (0.57, 2.4)	0.702
Laboratory confirmed malaria	2	(4.0)	11	(2.2)	1.9 (0.20, 9.0)	0.659

CI = confidence interval; OR = odds ratio.

* Data not available for all participants for all variables; % reflects the accurate denominator.

† Categories not mutually exclusive.

‡ Other - artisan, driver, guard, healthcare worker, manual laborer, miner, office worker, police, student, teacher, unemployed.

§ Pastoralist tribe - Barabaig or Maasai.

¶ Reference category in regression analysis.

**Figure 2.**
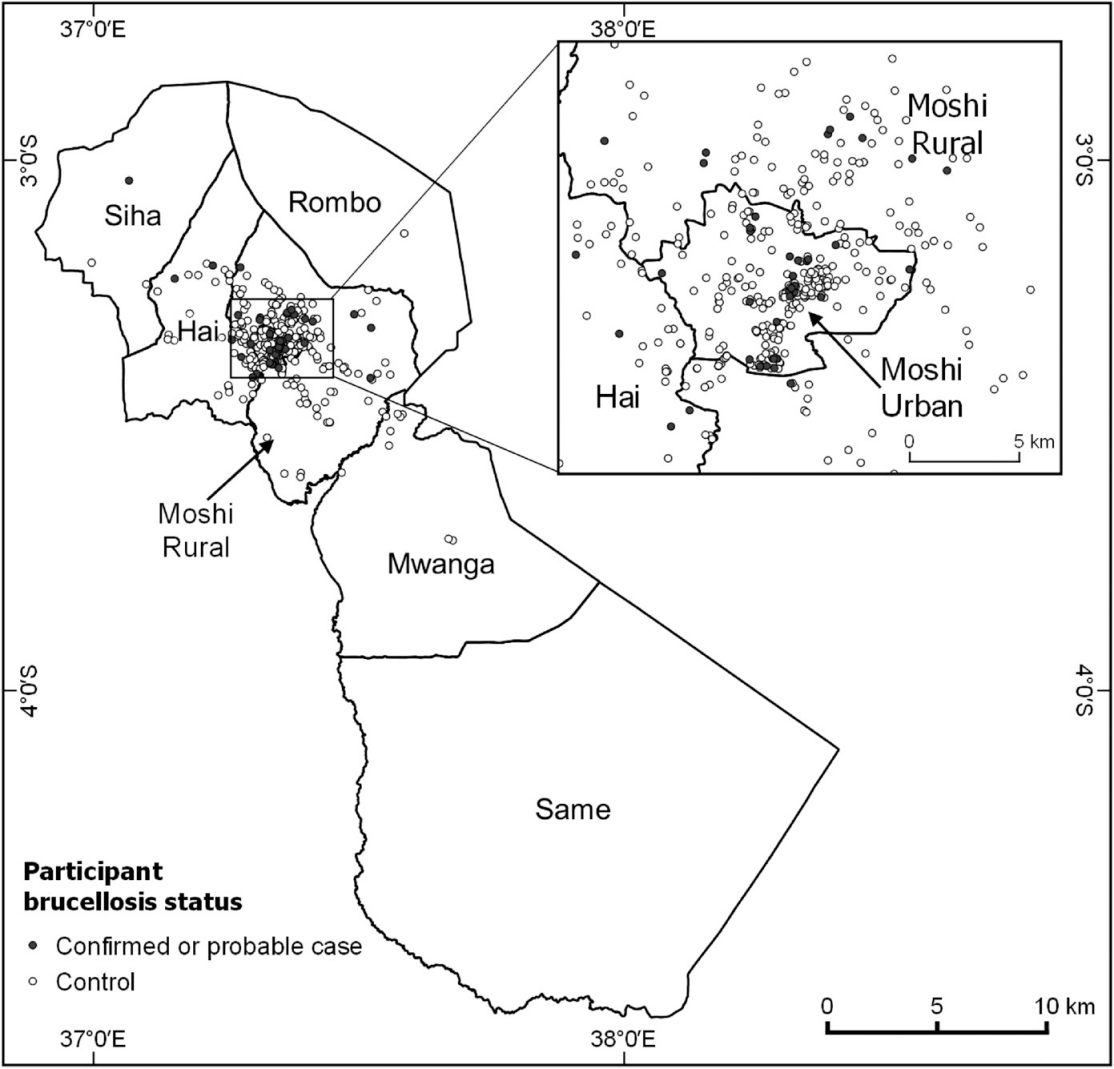
Location by district of participants with and without brucellosis, Kilimanjaro Region, northern Tanzania, 2012–2014.

Three hundred and ninety-four (70.1%) participants were over the age of 5 years. Older age was associated with brucellosis (OR 1.5 per year increase in age, CI 1.1, 2.0). Eight (1.4%) were from a pastoralist tribe. One hundred and five (18.7%) participants were farmers, and this was the most commonly reported livestock-related profession. No livestock-related occupation was associated with brucellosis. For 273 (48.6%) participants, the self-reported district of residence was Moshi Urban District. Geospatial coordinates were available for 474 (84.3%) participant households. Of those, 120 (25.3%) were in a rural zone, 97 (20.5%) were in a peri-urban zone, and 257 (54.2%) were in an urban zone. No specific zone was associated with brucellosis. There was no evidence of clustering in the spatial distribution of cases. Brucellosis cases and controls are mapped in Figure 2.

### Exposure scales.

To derive the livestock contact scale and the livestock blood exposure scale, we used the weights assigned to behaviors by eight and six internally consistent experts, respectively. Table 2 shows the variable weights that comprised each exposure scale. In the livestock contact scale, seeing livestock around the house had the lowest weight (0.65), whereas slaughtering livestock had the highest weight (4.19). In the livestock blood exposure scale, assisting livestock abortions had the lowest weight (0.87), whereas consuming raw livestock blood had the highest weight (8.52).

**Table 2:** Variables included in the exposure scales for participants with and without brucellosis, northern Tanzania, 2012–2014

Livestock contact scale		Livestock blood exposure scale	
Variable	Weight	Variable	Weight
See livestock around house	0.65	Assist livestock abortions	0.87
Keep livestock around house	0.84	Touch livestock carcass	1.25
Assist livestock abortions	1.15	Veterinarian	1.53
Feed livestock	1.15	Assist livestock births	1.82
Clean livestock waste	1.18	Slaughter livestock	6.02
Keep livestock inside house	1.56	Consume raw livestock blood	8.52
Livestock attendant	1.57	–	–
Assist livestock births	1.65	–	–
Veterinarian	2.18	–	–
Milk livestock	2.35	–	–
Slaughter livestock	4.19	–	–

### Univariable analysis.

Univariable regression results for behaviors and exposure scores on brucellosis are presented in Table 3. A number of livestock-related activities were associated with brucellosis, including assisting cattle births (OR 10.3, 95% CI 0.13, 820.1), assisting goat or sheep births (OR 7.2, 95% CI 1.5, 32.0), cleaning cattle waste (OR 4.1, 95% CI 1.6, 9.8), cleaning pig waste (OR 5.5, 95% CI 1.4, 18.8), feeding livestock (OR 2.8, 95% CI 1.4, 5.5), and consuming raw blood of livestock (OR 2.7, 95% CI 1.1, 6.3). Contact with livestock was associated with brucellosis (OR 1.2 per point increase in score, 95% CI 1.0, 1.3), as was contact with each individual livestock species. Contact with livestock blood was associated with brucellosis (OR 1.1 per point increase in score, 95% CI 1.0, 1.2), but contact with the blood of each individual livestock species was not.

**Table 3:** Univariable analysis of behaviors and exposures for participants with and without brucellosis, northern Tanzania, 2012–2014

	With brucellosis (*N* = 50)*		Without brucellosis (*N* = 512)*			
	*n*	(%)	*n*	(%)	OR (95% CI)	*P* value
Activities with livestock†						
Assist livestock abortions	3	(6.0)	12	(2.3)	2.7 (0.46, 10.3)	0.285
Cattle	0	(0.0)	2	(0.4)	–	–
Goats or sheep	1	(2.0)	9	(1.8)	1.1 (0.03, 8.5)	1.000
Pigs	2	(4.0)	1	(0.2)	21.0 (1.1, 1,258.3)	0.044
Assist livestock births	4	(8.0)	7	(1.4)	6.2 (1.3, 25.6)	0.023
Cattle	1	(2.0)	1	(0.2)	10.3 (0.13, 820.1)	0.040
Goats or sheep	4	(8.0)	6	(1.2)	7.2 (1.5, 32.0)	0.000
Pigs	0	(0.0)	0	(0.0)	–	–
Clean livestock waste	9	(18.0)	47	(9.2)	2.2 (0.87, 4.9)	0.097
Cattle	9	(18.0)	26	(5.1)	4.1 (1.6, 9.8)	0.000
Goats or sheep	4	(8.0)	30	(5.9)	1.4 (0.34, 4.2)	0.545
Pigs	5	(10.0)	10	(2.0)	5.5 (1.4, 18.8)	0.001
Feed livestock	16	(32.0)	74	(14.5)	2.8 (1.4, 5.5)	0.001
Cattle	14	(28.0)	46	(9.0)	3.9 (1.8, 8.1)	0.000
Goats or sheep	11	(22.0)	53	(10.4)	2.4 (1.1, 5.2)	0.013
Pigs	5	(10.0)	8	(1.6)	1.0 (1.7, 25.3)	0.000
Herd livestock	4	(8.0)	17	(3.3)	2.5 (0.59, 8.2)	0.374
Cattle	2	(4.0)	7	(1.4)	3.0 (0.30, 16.3)	0.214
Goats or sheep	4	(8.0)	17	(3.3)	2.5 (0.59, 8.2)	0.214
Keep livestock around house	15	(30.0)	174	(34.0)	0.83 (0.41, 1.6)	0.690
Cattle	12	(24.0)	117	(22.9)	1.1 (0.49, 2.2)	0.972
Goats or sheep	11	(22.0)	138	(27.0)	0.76 (0.34, 1.6)	0.566
Pigs	0	(0.0)	0	(0.0)	–	–
Keep livestock in house	8	(16.0)	45	(8.8)	2.0 (0.75, 4.6)	0.170
Cattle	2	(4.0)	4	(0.8)	5.3 (0.46, 37.8)	0.185
Goats or sheep	1	(2.0)	6	(1.2)	1.7 (0.04, 14.6)	0.962
Pigs	6	(12.0)	39	(7.6)	1.6 (0.54, 4.2)	0.401
Milk livestock	3	(6.0)	15	(2.9)	2.1 (0.38, 7.9)	0.419
Cattle	3	(6.0)	15	(2.9)	2.1 (0.38, 7.9)	0.419
Goats or sheep	0	(0.0)	2	(0.4)	–	–
Own livestock	19	(38.0)	195	(38.1)	1.0 (0.52, 1.9)	0.991
Cattle	15	(30.0)	125	(24.4)	1.3 (0.65, 2.6)	0.383
Goats or sheep	13	(26.0)	148	(28.9)	0.86 (0.41, 1.7)	0.664
Pigs	6	(12.0)	39	(7.6)	1.7 (0.54, 4.2)	0.276
See livestock around house	41	(83.7)	421	(82.4)	1.1 (0.49, 2.8)	1.000
Cattle	39	(79.6)	342	(66.8)	4.1 (1.6, 9.8)	0.067
Goats or sheep	38	(77.6)	379	(74.2)	1.4 (0.34, 4.2)	0.604
Pigs	15	(30.6)	165	(32.4)	5.5 (1.4, 18.8)	0.803
Slaughter livestock	7	(14.0)	53	(10.4)	1.4 (0.51, 3.4)	0.553
Cattle	6	(12.0)	42	(8.2)	1.5 (0.50, 3.9)	0.492
Goats or sheep	3	(6.0)	21	(4.1)	1.5 (0.27, 5.3)	0.526
Pigs	0	(0.0)	5	(1.0)	–	–
Consume livestock products						
Boiled or pasteurized milk	37	(74.0)	397	(77.7)	0.82 (0.41, 1.7)	0.660
Boiled or pasteurized dairy products	2	(4.0)	72	(14.1)	0.25 (0.03, 1.0)	0.052
Raw milk	0	(0.0)	2	(0.4)	–	–
Raw dairy products, total	12	(24.5)	107	(20.9)	1.2 (0.56, 2.5)	0.673
Cream	0	(0.0)	3	(0.6)	–	–
Butter	0	(0.0)	5	(1.0)	–	–
Cheese	0	(0.0)	0	(0.0)	–	–
Yogurt	12	(24.5)	99	(19.4)	1.4 (0.62, 2.8)	0.492
Other	0	(0.0)	1	(0.2)	–	–
Raw livestock blood	9	(18.0)	38	(7.4)	2.7 (1.1, 6.3)	0.033
Cattle blood	7	(14.0)	35	(6.8)	2.2 (0.78, 5.5)	0.067
Goat or sheep blood	3	(6.0)	9	(1.8)	3.6 (0.60, 14.9)	0.165
Pig blood	1	(2.0)	3	(0.6)	3.5 (0.06, 43.9)	0.624
Exposure scales						
Livestock contact, mean score (range)‡	0.64 (0, 12.67)	n/a	0.64 (0, 11.54)	n/a	1.2 (1.0, 1.3)	0.006
Cattle contact	0.64 (0, 10.56)	n/a	0.64 (0, 11.26)	n/a	1.2 (1.1, 1.4)	0.002
Goat or sheep contact	0.64 (0, 10.28)	n/a	0.64 (0, 9.29)	n/a	1.2 (1.0, 1.4)	0.019
Pig contact	0.00 (0, 7.64)	n/a	0.00 (0, 9.11)	n/a	1.2 (1.0, 1.5)	0.033
Livestock blood exposure	0.00 (0, 10.28)	n/a	0.00 (0, 10.28)	n/a	1.1 (1.0, 1.2)	0.037
Cattle blood	0.00 (0, 10.28)	n/a	0.00 (0, 10.28)	n/a	1.1 (0.99, 1.2)	0.086
Goat or sheep blood	0.76 (0, 10.28)	n/a	0.36 (0, 10.28)	n/a	1.1 (0.99, 1.3)	0.076
Pig blood	0.23 (0, 4.26)	n/a	0.11 (0, 7.27)	n/a	1.2 (0.87, 1.57)	0.298

CI = confidence interval; OR = odds ratio.

* Data not available for all participants for all variables; % reflects the accurate denominator.

† Livestock - cattle, goats, pigs, and sheep.

‡ Mean score (range), rather than *n* (%), is presented for all exposure scales.

### Multivariable analysis.

A multivariable model was constructed to explore independent associations between brucellosis and contact with each livestock species, contact with the blood of each livestock species, assisting births of each livestock species, consumption of raw dairy or dairy products, and consumption of boiled or pasteurized dairy and dairy products. Results are shown in Table 4. Of 562 participants, two (0.36%) had missing values for variables in the multivariable model and were dropped from the multivariable analysis. We controlled for age and for reported district of residence. Brucellosis was associated with assisting goat or sheep births (OR 5.9, 95% CI 1.4, 25.2) and with cattle contact (OR 1.2 per point increase in score, 95% CI 1.0, 1.4). Consuming boiled or pasteurized dairy products was identified as a protective factor (OR 0.12, 95% CI 0.02, 0.91). While consumption of raw dairy products was not associated with brucellosis (OR 0.89, CI 0.43, 1.9), model fit was better with inclusion of this variable.

**Table 4:** Multivariable analysis of characteristics of participants with and without brucellosis, northern Tanzania, 2012–2014

Variable*	OR	(95% CI)	*P* value
Assist sheep or goat births	5.9	(1.4, 25.2)	0.015
Age	1.5	(1.1, 2.0)	0.007
Cattle contact	1.2	(1.0, 1.4)	0.016
Consume boiled or pasteurized dairy products	0.12	(0.02, 0.91)	0.040
Residence outside Moshi Urban District	0.57	(0.29, 1.1)	0.086
Consume raw dairy products	0.89	(0.43, 1.9)	0.764

CI = confidence interval; OR = odds ratio.

* Variables originally included–age, assist livestock births, assist cattle births, assist goat or sheep births, assist pig births, livestock blood contact, cattle blood contact, goat or sheep blood contact, pig blood contact, livestock contact, cattle contact, goat or sheep contact, pig contact, consume raw dairy, consume raw dairy products, consume boiled or pasteurized dairy, consume boiled or pasteurized dairy products, district of residence.

## DISCUSSION

Our results point to multiple potential transmission pathways involving several livestock species in the epidemiology of human brucellosis in northern Tanzania. We showed that assisting goat or sheep births and contact with cattle were risk factors for brucellosis. We found that consuming boiled or pasteurized dairy products was protective. We also confirmed that brucellosis remains underdiagnosed by healthcare workers.

The association between assisting goat or sheep births and risk for brucellosis is consistent with the localization of *Brucella* to the reproductive tract of livestock. Indeed, other studies from Tanzania have demonstrated a relationship between exposure to livestock reproductive tract secretions and brucellosis or *Brucella* antibody seropositivity.^17,18,27^ Our finding that the riskiest behavior was assisting goat or sheep births is consistent with an analysis of human and livestock serologic data from northern Tanzania, which showed that human *Brucella* antibody seropositivity was more likely associated with goat and sheep contact than with cattle contact.^28^

We found evidence of an association between brucellosis and cattle contact. This is in agreement with other studies from sub-Saharan Africa that have implicated cattle as an important reservoir,^17,29,30^ and it indicates a potential role for cattle in the epidemiology of brucellosis in northern Tanzania. Our findings that brucellosis is associated both with cattle contact and with assisting goat or sheep births may suggest that both *B. abortus* and *B. melitensis* are circulating in northern Tanzania, or that a single *Brucella* species is infecting multiple livestock species.

While livestock-related occupations have been reported as risk factors for brucellosis in studies from sub-Saharan Africa,^7,31^ we did not observe an association between brucellosis and being a butcher, farmer, livestock attendant, or veterinarian. However, our data showed that activities involving livestock were not restricted to those who reported having livestock-related occupations. This highlights the importance of assessing specific behavioral risk factors rather than using proxies of risk, such as occupation or demographics.

We controlled for age in our multivariable model and observed that age was also an independent risk factor for brucellosis. We previously showed that increasing age was a risk factor for brucellosis in northern Tanzania,^32^ and older age has been identified as a risk factor for *Brucella* antibody seropositivity.^16,33,34^ These findings may be related to cumulative livestock exposure over time or to livestock-oriented activities that children do not perform.

While consumption of raw milk has been identified as a source of urban brucellosis in Uganda,^35^ we found no association between brucellosis and consumption of raw dairy or dairy products. One possible explanation is that the predominant livestock reservoir species in northern Tanzania is not the species from which people obtain most of their dairy. Interestingly, we observed a protective effect of boiled or pasteurized dairy product consumption. This could be due to the direct effect of better nutrition and health status in people who consume more boiled dairy products or reflect the effects of unobserved factors that are linked both with consumption of boiled dairy products and risk of brucellosis.

None of our 50 laboratory-confirmed brucellosis cases received a clinical diagnosis of brucellosis or effective brucellosis treatment during hospitalization. Several studies have shown limited healthcare provider awareness of zoonoses in Tanzania.^36–38^ Others have shown that despite the prevalence of endemic bacterial zoonoses such as brucellosis, clinicians overlook these diseases and over-diagnose malaria,^15^ as did the healthcare providers for our study participants. For every one laboratory-confirmed diagnosis of malaria, approximately 10 times that many participants were assigned a clinical diagnosis of malaria.

Our study had several limitations. We used self-reported district of residence in our analysis because of incomplete GPS data. Recall bias may have influenced participant responses about activities performed over the past month. The high ratio of independent variables to cases may have made our analysis underpowered to detect associations between brucellosis and individual behaviors. Most study participants were from urban and peri-urban zones, limiting our ability to assess brucellosis risk among rural dwellers. All brucellosis cases were diagnosed by serology rather than by culture, preventing analysis at the *Brucella* species level. Nearly one-quarter of participants with *Brucella* antibodies had titers too high to be considered controls and too low to be considered cases. It was difficult to draw epidemiologic conclusions from those participants, as they may have been exposed to *Brucella* in the past, had active disease but failed to mount a substantial antibody responses, or tested positive for *Brucella* antibodies due to cross-reactions between antibodies to other gram-negative bacteria and *Brucella* test antigens.^1^ While the exclusion of such participants, along with participants missing convalescent titers, may have influenced our outcomes, an exploratory analysis showed no significant differences between included and excluded participants in terms of age, sex, tribe, household location, consumption of raw dairy, consumption of livestock blood, or birthing livestock. And finally, our selection of behaviors to include in the exposure scales may not have been sufficiently comprehensive or may have been too exclusive. While we acknowledge that there is scope to improve the development of such exposure scales and to validate them, we believe that grouping data using biologic plausibility, rather than purely statistical methods, offered several advantages. Most our participants engaged in multiple potentially risky behaviors, and our methods offered a way to tease out epidemiologically meaningful behavioral patterns. In addition, we were able to evaluate the risk of exposures to potentially infectious sources even though we were unable to directly measure those exposures in our questionnaire.

In summary, we identified risk factors for human brucellosis in northern Tanzania. Knowledge of these risk factors may contribute to disease prevention and control efforts and may assist clinicians with risk stratification. Our research could be extended in a number of ways. To help target provision of education and personal protective equipment in northern Tanzania, levels of exposure to potentially infectious livestock body fluids could be quantified through bioaerosol sampling, detailed observation of livestock-related activities, and in-depth interviews. To develop livestock brucellosis vaccination strategies for northern Tanzania, bacterial isolates from human and livestock cases are needed to identify infecting *Brucella* species. Because pastoralists are more likely to have higher levels of exposure to livestock than nonpastoralists, it would be useful to repeat our study in a pastoralist context. In the meantime, the use of personal protective equipment among those with high levels of livestock contact, especially during the livestock birthing process, may help reduce disease transmission. Education efforts to promote boiling of milk and dairy products sold in urban areas may also help prevent disease. Finally, improving clinician awareness that not all fevers are malaria and strengthening diagnostic services for nonmalaria fever would improve the recognition and appropriate management of patients with brucellosis.
